# Understanding the tumor microenvironment for personalized immunotherapy in early-onset head and neck squamous cell carcinoma

**DOI:** 10.3389/fimmu.2024.1522820

**Published:** 2025-01-02

**Authors:** Yidan Shan, Di He, Fengguo Yan, Weijia Huang

**Affiliations:** Department of Oral & Maxillofacial Surgery, Second Affiliated Hospital, Zhejiang University School of Medicine, Hangzhou, China

**Keywords:** head and neck squamous cell carcinoma, early-onset, young, tumor microenvironment, immunotherapy

## Abstract

Early-onset head and neck squamous cell carcinoma (HNSCC) has been increasingly observed in recent years, exhibiting distinct tumor behavior and a unique tumor microenvironment (TME) compared to older age groups. Studies suggest that early-onset HNSCC is associated with specific risk factors and prognostic outcomes, while the underlying mechanisms driving these age-related differences remain unclear. In this review, we systematically examined original studies involving young HNSCC patient samples, focusing on the characteristics of the TME and potential for personalized immunotherapy. While further evidence is needed, our findings indicate that the TME in early-onset HNSCC often exhibits higher aggressiveness and immune suppression. Consequently, tailored immunotherapy may offer a promising therapeutic strategy for this distinct patient population.

## 1 Introduction

Cancer remains a leading cause of morbidity and mortality worldwide, imposing a substantial burden on global health (1, 2). Although cancer predominantly affects individuals aged>50 years, recent years there has been a notable increase in early-onset cancers (diagnosed in individuals aged<50 years) across various regions (3). This increase in early-onset cancers has far-reaching consequences on individuals and society, adding the overall disease burden (4, 5). Moreover, the adverse effects of cancer treatments at a younger age may result in long-term health complications, further worsening the burden associated with early-onset cancers (6).

Similarly, the incidence of early-onset head and neck squamous cell carcinoma (HNSCC) is increasing. HNSCC accounts for approximately 6% of all cancers globally and is ranked the sixth most common cancer (7, 8). Each year, approximately 900,000 new cases of head and neck cancers are diagnosed worldwide, with over 400,000 deaths annually (9).

While most HNSCC cases are diagnosed in older individuals (median age of 65 years) (10), the incidence among younger patients has increased, particularly in Asia, with marked increases in oral and oropharyngeal squamous cell carcinomas, particularly tongue and tonsil cancers (7, 11, 12). Traditional risk factors for HNSCC include tobacco and alcohol use; however, additional etiologies include Epstein-Barr virus (EBV) infection in the nasopharynx and human papillomavirus (HPV) infection in the oropharynx (13, 14). In Southeast Asia, the increasing use of betel nuts and the rising prevalence of HPV infections among younger populations have contributed to the growing incidence of HNSCC in this demographic (11, 15). Studies indicate that young patients with HNSCC (typically defined as individuals aged ≤30 to ≤45, accounting for 1%–8% of all HNSCC cases) exhibit distinct disease characteristics and progression patterns compared to older patients (16, 17); however, the mechanisms underlying these differences and the associated potential for targeted treatment remain unclear.

Emerging evidence suggests that younger cancer patients with cancer, including those with HNSCC, may present with unique biological and tumor microenvironmental (TME) characteristics. The TME comprises diverse cellular components and molecular signals, including immune cells, fibroblasts, endothelial cells, and various cytokines (18, 19), exhibiting distinct features in terms of inflammatory responses, immune evasion, and microenvironmental regulation. Studies have shown that the TME in younger patients may contain elevated levels and activity of immunosuppressive cells, such as regulatory T cells and myeloid-derived suppressor cells (20, 21), or a higher proportion of programmed death ligand 1 (PD-L1) expressing antigen presenting cells (APCs) (22), which could influence their response to immunotherapy. Therefore, exploring the unique TME in young patients with HNSCC is essential for understanding its underlying pathogenesis and to laying the groundwork for future, targeted therapeutic approaches.

In this study, we reviewed the current findings of the TME in early-onset HNSCC patients. The characteristics of the included studies were carefully presented and assessed, and the relevant findings (including TME profile and correlation factors) were summarized. Consequently, the potential immunotherapy for this age group was also discussed.

## 2 Materials and methods

Literature containing early-onset or young HNSCC in its the title or abstract from the last 20 years up to the end of October 2024) was retrieved through searching PubMed, Web of Science Core Collection, MEDLINE and Embase.

Inclusion criteria: Original articles investigating the characteristics of early-onset HNSCC.

Exclusion criteria: Review, meeting abstract, original research or bioinformatic analysis without real pathological specimen data, and literature not in English.

The exact search strategy, results and selection flow chart are presented in the **Supplementary Materials**.

## 3 Results

### 3.1 Characterization of included studies

**Figure 1:**
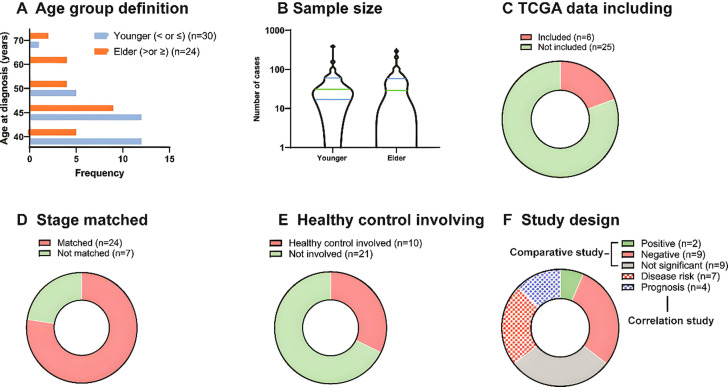
Characterization of included studies. **(A)** The frequency of age group definition of included studies. **(B)** The sample size of younger and older patient groups. **(C)** TCGA data including of included studies. **(D)** TNM stage match of the included studies. **(E)** Healthy (blank) control involving of the included studies. **(F)** Study design of included studies. There were 20 comparative studies and 11 correlation studies in total, and 2, 9, 9 studies reported positive, negative and not significant of younger patients compared to older ones. And 7 studies presented disease correlation and 4 presented prognostic correlation.

After the screening, 31 studies were included in the final analysis. Different definitions of ‘younger’ and ‘older’ patients groups were observed (**Figure 1A**). Twenty-four studies defined younger patients as those aged<40 years (n=12) or <45 years (n=12), five studies defined younger patients as those aged<50 years, and one study defined younger patients as those aged<70 years. Studies defined the older group as follows: patients >45 years old; patients aged 40 (n=5), 50 (n=4), and 60 years (n=4); and patients aged >70 years (n=2).

The sample sizes also varied (**Figure 1B**). Nine studies did not include an older group, and the number of younger and older patients ranged from 4 to 386 and 4 to 294, respectively, with a relatively similar median. Among the 31 studies, 6 included The Cancer Genome Atlas (TCGA) data (**Figure 1C**), 24 matched TNM stages between patient groups (**Figure 1D**), and 10 used healthy or blank controls for comparison with the study groups (**Figure 1E**). Finally, among 20 comparative studies, 2 studies showed that younger groups were positive for tumor aggressiveness compared to older groups, 9 showed negative results, and 9 showed non-significant results. In addition, among 11 correlation studies, 7 presented disease risk and 4 prognosis correlations (**Figure 1F**).

### 3.2 Possible TME of early-onset HNSCC

**Figure 2:**
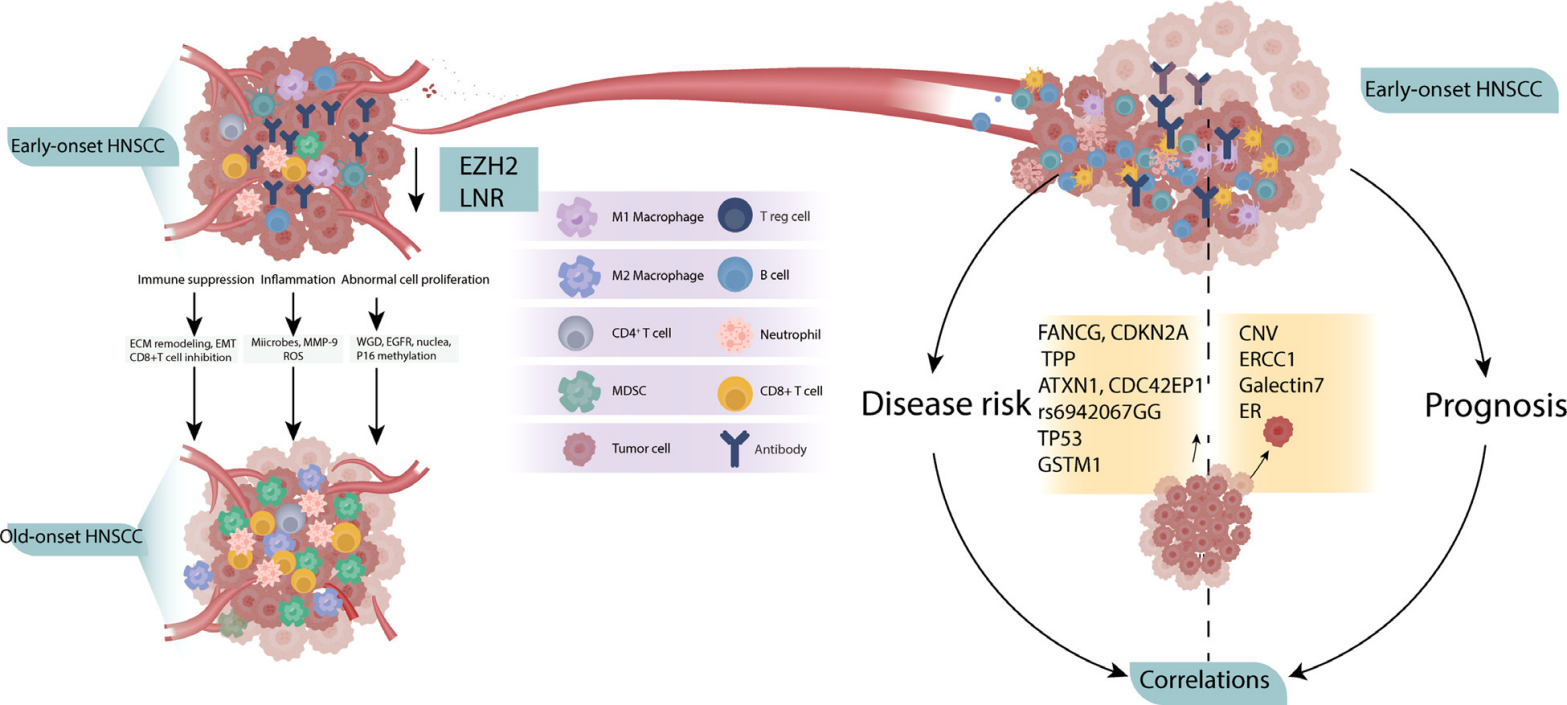
Possible TME of early-onset HNSCC. Compared to old-onset, the early-onset group presented a more aggressive TME (left), with independent correlation studies showing potential disease risk and prognosis (right).

Among the 31 studies included in the final analysis, 20 reported comparisons between patients with early- and late- onset HNSCC, and 11 reported potential correlations between specific factors and early onset. The possible TME based on these studies are shown in **Figure 2**. The two positive results showed that young patients presented a less harmful TME than older patients. These include lower expression of enhancer of zeste homolog 2 (EZH_2_) (23) and lower neutrophil-to-lymphocyte ratio (NLR) (24) in younger patients, reflecting lower invasion and metastasis of the tumor behavior in this age group. However, the remaining nine studies showed opposing results, indicating that younger tumors have a more aggressive TME. Increased extracellular matrix (ECM) remodeling, epithelial-to-mesenchymal transition (EMT), and a decreased quantity of CD8+T cells lead to a more immunosuppressive TME (20). The enrichment of inflammatory microbes, increased levels of reactive oxygen species (25) and MMP-9 expression (26) result into inflammatory TME. In addition, the increased WGD (27), EGFR level (26, 28, 29), nuclear polymorphism and mitotic index (30), and P16 methylation leads to abnormal cell proliferation and invasion (31).

Correlation studies have identified early-onset factors for both disease and prognosis. The following factors have been associated with HNSCC risk in young patients: major histocompatibility complex class I-related chain A (MICA) A5.1 homozygous genotype (32), germline variants in FANCG, CDKN2A and TPP (33), drive genes ATXN1 and CDC42EP1 (34), rs6942067 GG genotype (35) in non-HPV and non-smokers, TP53 variation (36), HPV16 positive (37) and GSTM1 null genotype (38) were reported to be associated with HNSCC risk in young patients. In addition, copy number variation (39), ERCC1 (40), galectins-7 (41) and estrogen hormone receptor expression (42) are associated with the prognosis to young HNSCC patients.

## 4 Discussion

### 4.1 Altered TME in young group

The evolving landscape of early-onset HNSCC demands a better understanding of host-tumor interactions in the TME to improve the effectiveness of immunotherapy. However, the impact of complex tumor-infiltrating immune cell profiles on responses to immune checkpoint inhibitors is not fully understood; limited evidence has yielded contradictory findings. Although only direct analysis of the TME was reported, the studies showed that early-onset HNSCC is distinct from average-onset terms of tumor behavior and prognosis, indicating different therapeutic demands.

According to Révész et al. (23) and Zhang et al. ‘s (24) studies, TME of younger patients presented more gently compared to older ones. EZH_2_ expression and NLR were lower in the young groups than in the older group. Having a well-defined oncogenic role in cancer initiation, progression, metastasis, metabolism, and drug resistance, and in the modulation of antitumor immunity in various cancers, EZH_2_ has been defined as an effective marker of the tumor aggressiveness and tumorigenic potential and plays essential roles in driving cancer cell immunoediting and as an immune escape regulator (43). Inhibition of EZH_2_ could suppress oral squamous cell carcinoma (OSCC) progression via modulate EZH_2_/Wnt/beta-catenin pathway, both *in vitro* and *in vivo* (44). Clinically, the use of EZH_2_ inhibitors in combination with IO represents a compelling strategy to remodel the TME, potentially overcoming immune evasion and enhancing therapeutic outcomes in breast cancer (16), mesothelioma (45), non-Hodgkin lymphoma (46) and other cancers.

Similarly, a low NLR is associated with reduced cancer invasion and metastasis of the cancer. When this is observed in young patients, a better prognosis is usually expected. In patients with muscle-invasive bladder cancer (MIBC), this is associated with increased CD3^+^ T cells and B cell infiltration, lead to improved response and long-term outcomes (47).

Genetic factors were reported to be associated with the occurrence of HNSCC or prognosis. The membrane protein MICA generate response to various cellular stresses such as infection and oncogenic transformation, its mechanism of A5.1 allele association with disease risk of young OSCC remained unclear. Current evidence shows it’s essential in etiology and immune response of cancer, both positive or negative. Li et al. reported MICA expression positively related to the CD8^+^ T cell infiltration in hepatocellular carcinoma (48), however Wu et al. found the releasing of MICA progressed tumor immune escape (49).

Cury et al. reported germline variants CDKN2A and RECQL4 are associated with young HNSCC (33). CDKN2A variant closely associated with weak expression of immune-inflammatory pathways in the TME, potentially leading to reduced immune cell activity and weakened immune responses. RECQL4 variant may play a critical role in tumorigenesis and progression by regulating immune responses. Also, these two variants played roles in immune infiltration and the interactions of chemokines and their receptors under immune cells in melanoma (50), and existed in pancreatic ductal adenocarcinoma but are not currently actionable targeted (51). Similarly, ATXN1 and CDC42EP1 have also been reported as driver genes in HNSCC; however, their relevance to young patients cannot be conclusively determined (34). On SNP level, rs6942067 GG genotype is significantly higher in young and in HPV negative non-smoking HNSCC than in other HNSCC, which associated with DCBLD1 expression (35, 52). In addition, TP53 variation, HPV16 positive and GSTM1 null genotype were also mentioned. Different studies also showed CNV, ERCC1 expression, galectin-7 and Estrogen related to prognosis in young HNSCC. Overall, distinct genotypes may either promote or inhibit tumor progression by influencing various components within the TME. In young HNSCC patients, the high expression of specific genotypes has been associated with immune microenvironment activation. In addition, CD8^+^ T cells are crucial effector cells in anti-tumor immune responses the decreased amount in TME suggested a heightened state of immune suppression. The diminished immune surveillance allows tumor cells to evade detection and destruction by the immune system, thereby promoting tumor growth and metastasis.

Furthermore, the marked acceleration in cell proliferation of younger patient indicated that tumor cells may exhibit dysregulation in the signaling pathways controlling proliferation. Such abnormal proliferation is often associated with imbalances in cell cycle regulation and disruptions in apoptotic mechanisms, further driving rapid tumor growth and dissemination (53, 54).

The crosstalk among cells in the TME plays essential roles in the development and progression of HNSCC. Although there is no direct evidence showing exact cellular crosstalk, it can be inferred that the early-onset group exhibits altered communication between immune cells (lymphocytes and CD8+ T cells) and tumor cells compared to the older group. Additionally, the different crosstalk induced by changes in cellular molecules (e.g., EZH_2_ protein) also plays an indirect role.

### 4.2 Heterogeneity of the studies

The included studies were highly heterogenous. The definition of younger groups had varying age ranges depending on different guidelines. The samples sizes of the young and older groups were relatively similar; however, large cohort data is lacking. Some studies involved supplementary data from TCGA, which increased the number of patients available for comparison; however, considering the characteristics of different ethnicities and nationalities, bias may exist. Most studies matched TNM stages between groups, while less involved healthy patients as blank controls. These variations contribute to the unreliability of the studies, complicating efforts to combine and compare results. To reduce heterogeneity and achieve more definitive conclusions, subgroup analyses and meta-analyses are recommended when sufficient data points are available.

### 4.3 Personalized immunotherapy tailoring based on current TME findings

Personalized immunotherapy emphasizes treatment to each patient’s unique tumor profile and immune response, significantly enhancing effectiveness and reducing side effects compared to standard approaches (55–57). To develop personalized immunotherapy for specific cohorts, understanding the TME is essential (58, 59). Although current evidence is limited in scale and fragmented, it can be concluded that immunotherapy for early-onset HNSCC patients should focus on targeting EGFR, inhibiting ECM remodeling and EMT, and paying attention to high P16 methylation and specific coexisting microbial infections.

Additionally, defined genetic risk factors, including variations in MICA A5.1, FANCG, CDKN2A, and TPP, as well as alterations in ATXN1, CDC42EP1, and TP53, offer potential therapeutic and preventive pharmacological targets. Furthermore, CNV, ERCC1, galectins-7, and ER expression are promising candidate predictive biomarkers. It can be learned that multi-dimensional approaches including blood test, immunohistochemistry, PCR, RNA-sequencing, whole exosome sequencing, microbiota and whole genome have been used from comparative and correlation studies.

For young patients, the focus should be on strategies that restore CD8+ T cell function, regulate associated genetic factors, and target immune escape mechanisms (such as MICA shedding). EZH_2_ inhibitors have shown potential in remodeling the TME and enhancing immune responses. Therefore, in young patients, if the immune suppression in the TME is low, the combination of EZH_2_ inhibitors with immunotherapy therapies may be particularly effective. For older patients, this combination therapy may also be effective, but the treatment regimen should be adjusted based on the specific TME characteristics.

### 4.4 Future works

Overall, based on the current findings, larger-scale clinical studies are necessary in the future to verify these results. Further investigations into TME cellular characteristics, such as EZH_2_, MICA expression, and NLR, would also provide valuable evidence. Additionally, developing new therapeutic targets and predictive biomarkers based on these findings and translating them into real clinical practice is anticipated.

## 5 Conclusion

In conclusion, early-onset HNSCC demonstrates unique TME characteristics, often marked by heightened aggressiveness and immune suppression compared to HNSCC in older patients. These findings highlight the need for further investigation into the specific mechanisms driving these age-related differences. Personalized immunotherapy provide potential as an effective therapeutic strategy for early-onset HNSCC, underscoring the importance of tailored approaches in addressing the distinct clinical and biological features of this patient cohort.
