# Supplementary material for: Understanding the tumor microenvironment for personalized immunotherapy in early-onset head and neck squamous cell carcinoma
Source: Front Immunol. 2025 Jan 2;15:1522820. doi: 10.3389/fimmu.2024.1522820 (PMC11739722; doi:10.3389/fimmu.2024.1522820)
Supplement: Supplementary file 1 [file DataSheet1.docx]

Supplementary Material

# Search strategies

("early-onset" OR "young adults" OR "adolescents" OR "early diagnosis" OR "age < 45") AND ("head and neck cancer" OR "head and neck neoplasms" OR "oral cancer" OR "pharyngeal cancer" OR "laryngeal cancer") AND ("characteristics" OR "risk factors" OR "clinical presentation" OR "genetic markers" OR "epidemiology" OR "prognosis" OR "molecular profile")

# Supplementary Figures and Tables

## Supplementary Figure


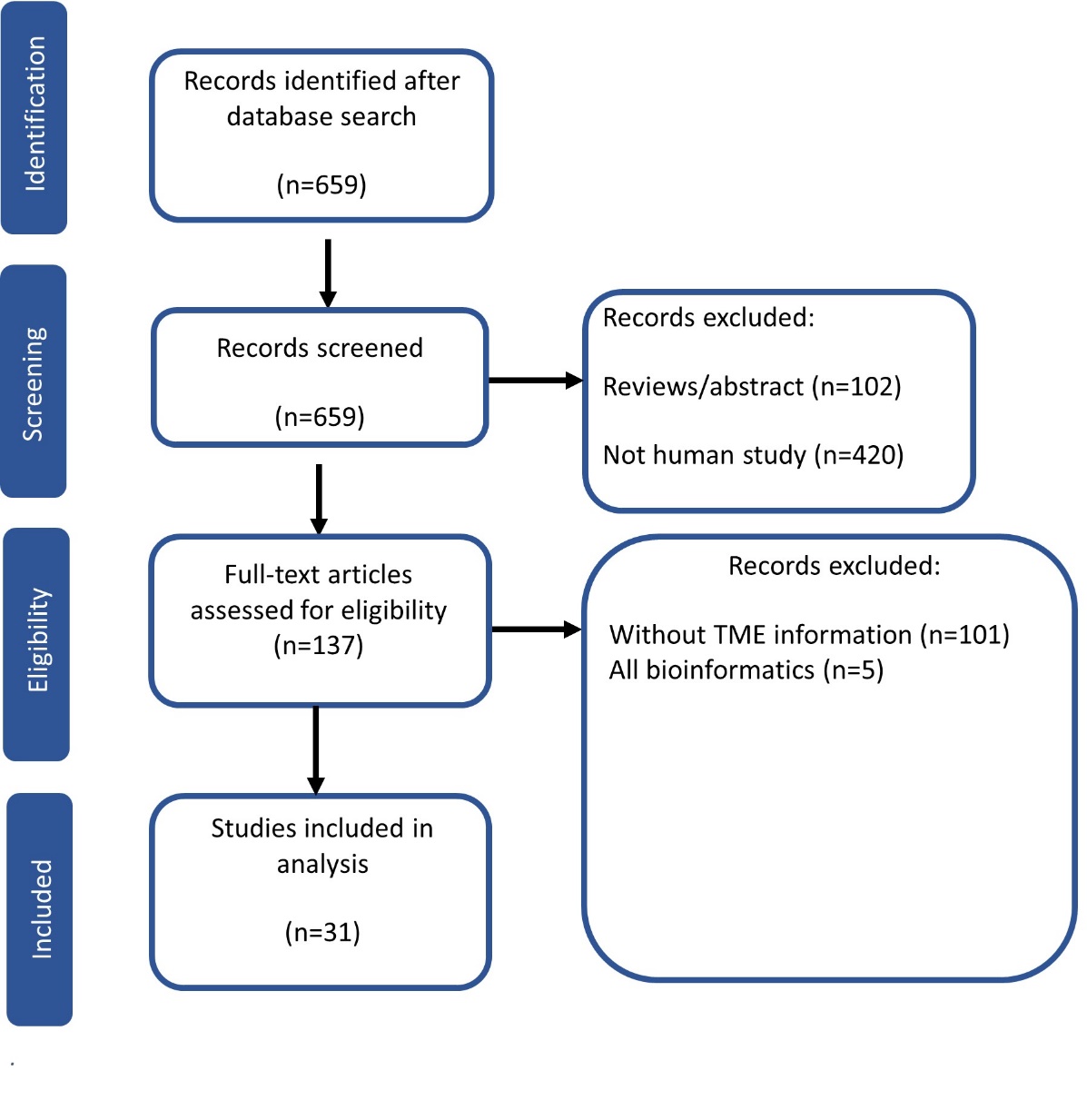


**Supplementary Figure 1.** Flow chart describing the process of article selection including exclusion criteria.

## Supplementary Table

Supplementary Table 1. Studies included for final analysis

| Author | Year | Definition of young | Definition of old | TNM match | TCGA data | Healthy control |
| --- | --- | --- | --- | --- | --- | --- |
| Révész et. al | 2024 | 39 | 63 | 1 | 0 | 0 |
| Estephan et. al | 2024 | 45 | 60 | 1 | 1 | 0 |
| Zhang et. al | 2022 | 50 | 60 | 1 | 0 | 0 |
| Sood et. al | 2022 | 45 | 45 | 0 | 0 | 0 |
| Satgunaseelan et. al | 2022 | 50 | N/A | 1 | 1 | 0 |
| Tani et. al | 2021 | N/A | N/A | 1 | 0 | 1 |
| Satgunaseelan et. al | 2021 | 50 | 50 | 0 | 1 | 0 |
| Molimard et. al | 2021 | 45 | 45 | 1 | 0 | 0 |
| Cury et. al | 2021 | 49 | N/A | 0 | 0 | 1 |
| Campbell et. al | 2021 | 50 | 50 | 1 | 1 | 0 |
| Jeske et. al | 2020 | 40-69 | 70 | 1 | 0 | 1 |
| Zhang et. al | 2019 | 40 | 60 | 1 | 0 | 0 |
| Teixeira et. al | 2019 | 40 | 65 | 1 | 0 | 0 |
| Gu et. al | 2019 | 40 | 60 | 1 | 1 | 0 |
| Gong et. al | 2019 | 40 | N/A | 1 | 0 | 1 |
| Cardin et. al | 2019 | 40 | 40 | 1 | 1 | 0 |
| Miranda et. al | 2018 | 40 | N/A | 1 | 0 | 1 |
| Costa et. al | 2018 | 40 | 50 | 1 | 0 | 1 |
| Miranda et. al | 2018 | 40 | 40 | 1 | 0 | 0 |
| Mesquita et. al | 2016 | 45 | N/A | 1 | 0 | 0 |
| Grimm et. al | 2016 | 45 | 45 | 1 | 0 | 1 |
| Braakhuis et. al | 2016 | 42 | N/A | 0 | 0 | 0 |
| Ur Rahaman and S. M. Ahmed Mujib, B | 2014 | 40 | 40 | 1 | 0 | 0 |
| Braakhuis et. al | 2014 | 45 | 45 | 1 | 0 | 0 |
| Kostrzewska et. al | 2013 | 45 | 45 | 1 | 0 | 1 |
| Pfeiffer et. al | 2011 | 45 | 45 | 1 | 0 | 0 |
| Farias et. al | 2010 | 45 | 45 | 1 | 0 | 1 |
| Chen et. al | 2010 | 40 | 40 | 0 | 0 | 1 |
| Su et. al | 2010 | 54 | 54 | 0 | 0 | 0 |
| Gawecki et. al | 2007 | 45 | 45 | 0 | 0 | 0 |
| Siriwardena et. al | 2007 | 40 | 50 | 1 | 0 | 0 |

*1=yes, 0=no.
